# Corpus callosum hematoma, as a rare complication of COVID‐19

**DOI:** 10.1002/ccr3.5178

**Published:** 2021-12-09

**Authors:** Mohammad Khair Hamad, Dima Hamed Takruri, Satya Narayana Patro, Mhd Baraa Habib, Vishwajit Verma

**Affiliations:** ^1^ Internal Medicine Department Hamad Medical Corporation Doha Qatar; ^2^ Family Medicine Department Primary Health Care Corporation Doha Qatar; ^3^ Neuroradiology Department University of Arkansas for Medical Sciences Little Rock Arkansas USA; ^4^ Intensive Care Department Hamad Medical Corporation Doha Qatar

**Keywords:** anticoagulation, corpus callosum hematoma, COVID‐19 pneumonia, pulmonary embolism

## Abstract

A 46‐year‐old gentleman had a complicated course of COVID‐19 pneumonia. Despite the recovery of the respiratory status, he developed corpus callosum hematoma and critical illness neuropathy/myopathy. The clinical situation became more complicated by developing pulmonary embolism that required anticoagulation. Fortunately, the patient made a good recovery.

## Graphical abstract

A rare site of intracranial bleeding—corpus callosum bleeding—complicates the course of COVID‐19 pneumonia and the complex management of pulmonary embolism in the presence of intracranial bleeding.

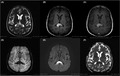

## 1 INTRODUCTION

SARS‐CoV‐2 is causing a worldwide pandemic that mainly affects the pulmonary system. However, other system involvement is being increasingly noticeable in COVID‐19 patients.^1^ Common neurological manifestations are anosmia, dysgeusia, and headache. Other serious complications, like stroke, are less commonly described. Intracerebral hemorrhage is rarely associated with COVID‐19.^2^ We present a COVID‐19 patient who has a complicated clinical course with challenging management.

## 2 CASE REPORT

A previously healthy 46‐year‐old male patient presented to the emergency department complaining of hotness, cough, and loss of smell for 4 days duration. On presentation, he was conscious, alert, and oriented to time, place, and person. He was febrile (temperature of 38.9°C), tachycardia with a heart rate of 104 bpm, tachypnoeic with a respiratory rate of 28 breaths per minute, blood pressure was 107/81 mmHg, and O_2_ saturation on room air was 97%.

On physical examination, pupils were equal in size and reacting to light. Chest auscultation showed clear breath sounds bilaterally with normal S1S2 and no murmurs. The abdomen was flat, soft, with no tenderness or guarding. Extremities were unremarkable, no bipedal edema, bilateral full, and equal pulses. The neurological examination showed Glasco coma scale (GCS) 15/15, and no neurologic deficits.

Blood investigations revealed WBC to be 5.7 × 10^9^ L, with low lymphocytic count 0.7 × 10^3^ µl, hemoglobin 15.3 g/dl, platelet count 122 × 10^9^ L, urea 3.04 mmol/L, creatinine 89 µmol/L, CRP 140 mg/L, and normal electrolytes.

Chest X‐ray demonstrated multiple pneumonitic patches seen in both lung fields, which were suggestive of viral pneumonia. Nasopharyngeal swab for COVID‐19 PCR was positive. Based on the work‐up as mentioned above, the patient was admitted as a case of COVID‐19 pneumonia. His condition deteriorated the next day of admission, and he was more tachypnoeic, requiring O_2_ supplementation by nasal cannula.

He was transferred to the medical intensive care unit (MICU) for observation. As his oxygen requirements increased, he was started on nonrebreathing mask then switched to noninvasive ventilation (continuous positive airway pressure). Despite this, his condition deteriorated further, requiring endotracheal intubation, and he was started on mechanical ventilation.

He received a treatment protocol for COVID‐19 infection according to our hospital protocol following the international guidelines at that time, which was as the following:

1. Hydroxy‐chloroquine 400 mg once daily ×10 days.
2. Azithromycin 500 mg ×7 days.
3. Methylprednisolone 40 mg IV q12 h for 5 days.
4. Tocilizumab 600 mg IV, total of two doses (8 days between the two doses).

He was kept on enoxaparin for deep vein thrombosis prophylaxis, and the dose was adjusted according to his clinical situation with monitoring of Anti Xa. Furthermore, he received convalescent plasma.

During this time, the platelet counts had dropped to as low as 92×10^3^ for 2 days then improved to normal levels. The INR was normal, and heparin‐induced thrombocytopenia test was negative. There were no noticeable overt bleeding episodes.

He was kept on mechanical ventilation, and the ventilator setting was adjusted. Proning was required three times, and his condition was gradually improving. Sedation and muscle relaxation were tapered off, and after 18 days, he was successfully extubated.

After extubation, the patient could not move his four limbs, but he had an intact level of consciousness. Physical examination revealed a power of 1/5 in all muscle groups of upper and lower limbs, bilateral lower limb wasting with hyporeflexia, and no fasciculations, the plantar reflex was negative, and the sensation was intact.

**FIGURE 1:**
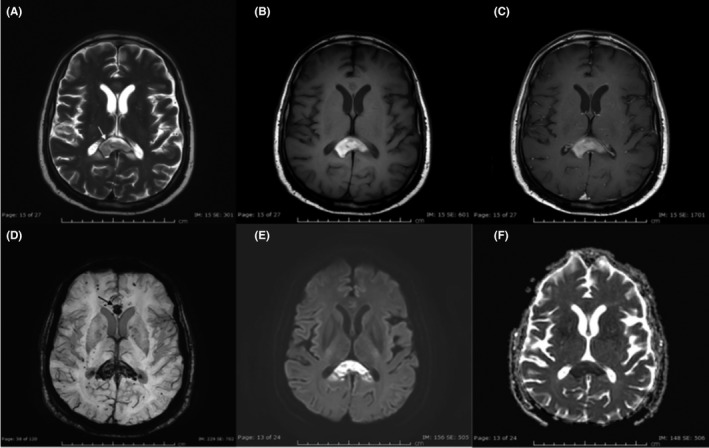
MRI brain axial T2W image of the brain (A) shows well‐defined hyperintense intraparenchymal hematoma in the splenium of the corpus callosum with mild perilesional vasogenic edema (white arrow). Pre‐ and post‐contrast axial T1W images of the brain (B and C) demonstrate hyperintense hematoma in the splenium of the corpus callosum with no post‐contrast enhancement. Axial susceptibility‐weighted image (SWI) of the brain (D) reveals blooming in the splenial hematoma and another hemorrhagic lesion in the genu of the corpus callosum (black arrow), and there are also multiple microhemorrhages in the cortical‐subcortical regions of bilateral cerebral hemispheres. Axial diffusion‐weighted image and corresponding ADC map of the brain (E and F) show diffusion restriction in the subacute splenial hematoma

As an evaluation of his quadriplegia, brain and spinal cord magnetic resonance imaging (MRI; Figure 1) was done and showed late subacute hematoma involving the corpus callosum, with a background of numerous supra and infratentorial foci of microbleeds. Cervicodorsal spinal cord MRI was unremarkable. Accordingly, enoxaparin was stopped on the same day.

The neurosurgery team was consulted regarding any possible therapeutic intervention for the subacute callosal hematoma. Since the hematoma was not causing any mass effect and the fact that it was subacute with no associated intraventricular hemorrhage, no neurosurgical intervention would benefit.

Neurology team evaluation suggested critical illness neuropathy/myelopathy as a cause of the quadriplegia rather than the hematoma itself. Due to isolation precautions, nerve conduction study nor electromyography could be done.

**FIGURE 2:**
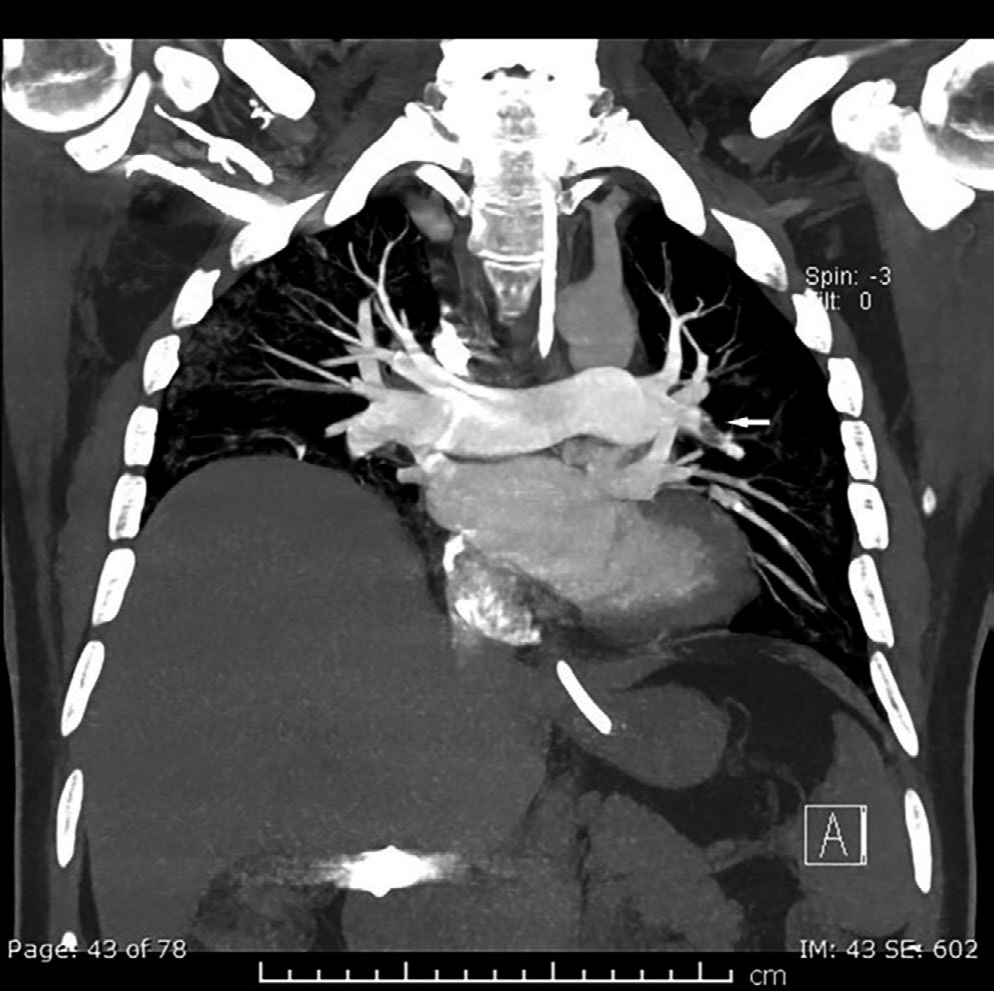
Coronal maximum intensity projection (MIP) image of pulmonary angiogram shows embolus (white arrow) in the anterior branch of the left main pulmonary artery

The next day, he was noted to have persistent tachycardia with HR ranging from 100 to 120 BPM. Computed tomography (CT) pulmonary angiogram (Figure 2) was done and showed two pulmonary thromboembolism. The pulmonary thromboembolism involved the anterior branch of the left main pulmonary artery extending to subsegmental branches and the posterior basal segmental and subsegmental branches of the right lower lobe pulmonary artery.

**FIGURE 3:**
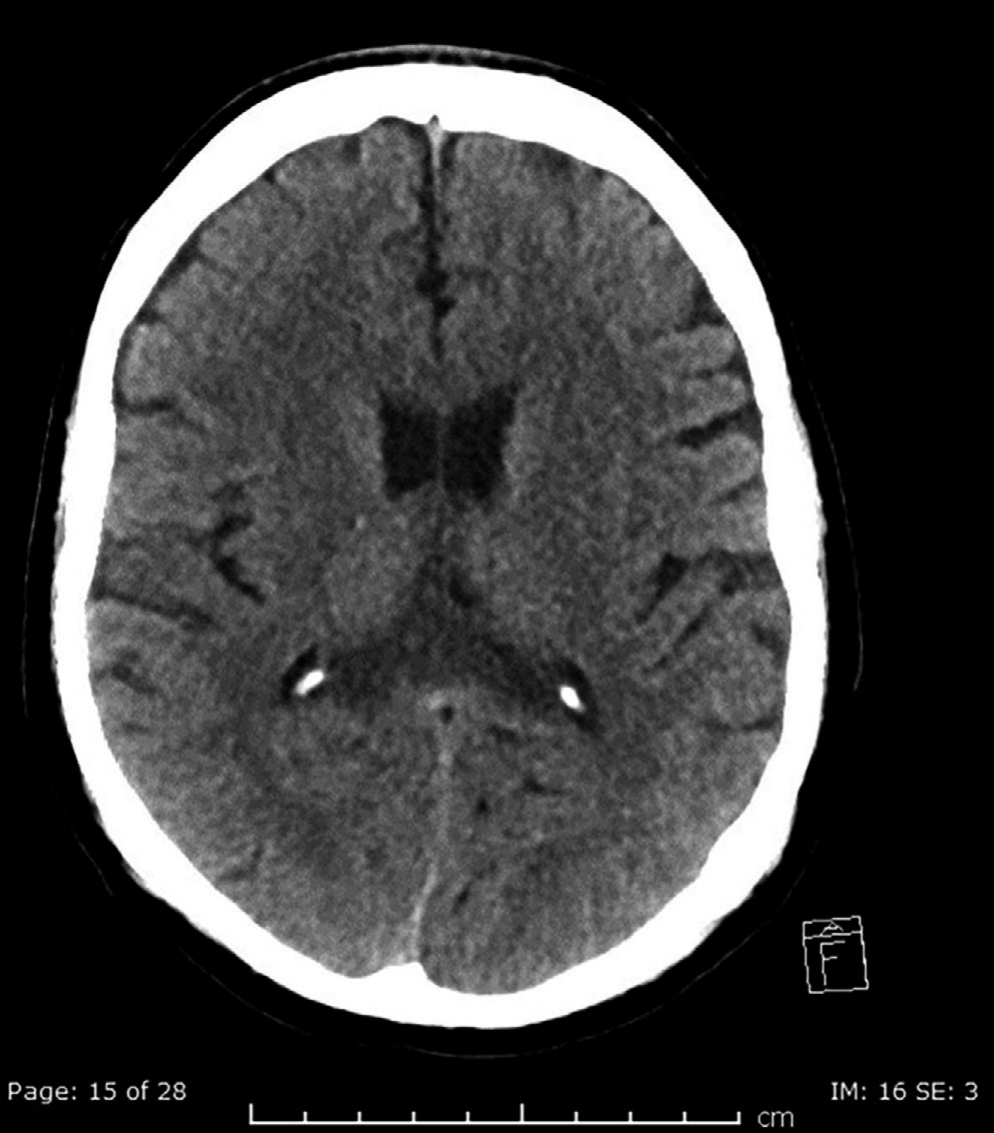
Follow‐up axial CT head image after 1 week shows resolution of the hematoma with residual hypodensity in the splenium

Considering the intracranial bleeding, he was commenced on heparin infusion with close monitoring of the neuro vitals. Then, he was switched to therapeutic low molecular weight heparin. Follow‐up head CT scan (Figure 3) demonstrated the same subacute corpus callosum hematoma, with no acute changes or bleeding.

COVID‐19 PCR swab was repeated twice, and both results were negative.

The patient was tapered off O_2_ supplementation, and he maintained an excellent O_2_ saturation on ambient air.

After discussing the case with the hematology team, considering the patient's condition, the patient was given a therapeutic dose of enoxaparin subcutaneously for 3 months as a case of provoked pulmonary thromboembolism.

Extensive physiotherapy sessions were started with gradual and noticeable improvement in the muscle group's power.

The patient was treated as an inpatient for 2 months and then continued physiotherapy in a rehabilitation institute for another 1 month. On discharge, muscle power was 4/5 and he was independent in most of the activities of daily living, with the need for assistance in using stairs only.

On further follow‐up, the patient continued to attend outpatient physiotherapy sessions weekly. He completed 3 months of enoxaparin for anticoagulation, with no reported bleeding episodes.

The patient is now functioning independently, and he returned to his work.

## 3 DISCUSSION

A novel coronavirus was first identified in China, causing human respiratory disease.^3^ As the virus is spreading and causing a worldwide pandemic, our understanding of the disease spectrum is evolving.

Accumulating evidence shows that SARS‐CoV‐2 is not only limited to the respiratory system but also has a deleterious effect on other organs and systems such as the cardiovascular, gastrointestinal, and nervous systems.^1^, ^4^

Both glial and neuronal cells express ACE2 receptors, making them susceptible to SARS‐CoV‐2 attack.^5^, ^6^, ^7^ The virus makes access to the brain either by the cerebral circulation, where ACE2 receptors are expressed in the endothelial lining of the cerebral vessels, or by the retrograde path from the olfactory bulb through the cribriform plate.^5^, ^6^, ^7^, ^8^

Both central and peripheral nervous system manifestations have been reported as COVID‐19 specific or related to critical illness.^2^ Disease spectrum may range from anosmia, ageusia, headache, and dizziness without other clinical features to more severe involvement such as encephalitis, acute disseminated encephalomyelitis, myelitis, cerebrovascular manifestations, and peripheral and muscle diseases.^2^

Cerebrovascular disease is an essential complication of COVID‐19, with a reported incidence of 2%–6% of hospitalized patients. The hypercoagulable state with the inflammatory cascade leading to endothelial damage predisposes to acute cerebrovascular events.^2^

Intracerebral hemorrhage is not a common event in general intensive care unit (ICU) patients. Hematologic malignancies, severe thrombocytopenia, sepsis complicated by renal and hepatic dysfunction, mechanical ventilation with high inspiratory pressures, and high CO_2_ could be associated with increased risk of intracranial hemorrhage (ICH) in critical illness.^9^

Few case reports and case series reported intracranial hemorrhage as a complication of COVID‐19,^4^, ^10^, ^11^, ^12^, ^13^, ^14^, ^15^, ^16^, ^17^, ^18^ while only one report showed that ICH happened before the respiratory manifestations.^4^

ACE2 receptors in the endothelium of cerebral vessels make them a target for SARS‐CoV‐2, which leads to endothelial dysfunction and dysregulation of local blood pressure and flow, resulting in vessel wall rupture.^12^, ^13^, ^17^ In a series of COVID‐19 nonsurvivors, postmortem brain MRI suggests vasculopathic changes that could be related to the viral damaging effect on the endothelium.^19^

In some reports, the intracerebral bleeding was secondary to the hemorrhagic transformation of cerebral stroke,^12^ hemorrhagic transformation of cerebral venous thrombosis,^18^ and meningoencephalitis complicated with ICH.^15^

In one small series of three patients, the cause of ICH was uncertain, either secondary to therapeutic anticoagulation or as a complication of COVID‐19. They also shared a common finding on brain imaging showing anoxic brain injury.^20^

Diffuse brain edema and multifocal hemorrhages support the speculation that anoxic brain injury and cytokine storm rather than anticoagulation led to ICH in COVID‐19 patients.^6^, ^7^, ^10^ In the case series of COVID‐19 nonsurvivors, brain postmortem histopathological examination revealed hypoxic injury without evidence of encephalitis.^21^

Spontaneous corpus callosum hematoma is rarely described in the literature.^22^ Possible causes are traumatic brain injury, hypertension, ruptured anterior communicating artery or pericallosal artery aneurysm, bleeding associated with tumors, or encephalitis,^23^ which was not the case in our patient who did not have any of the risk factors.

One series of 11 patients describes diffuse leukoencephalopathy with microhemorrhages. In 4/11 patients, the location of the microhemorrhages was in the corpus callosum, and all patients were on mechanical ventilation and monitored anticoagulation, but there was no bleeding elsewhere in the body, and brain hypoxia was proposed as the mechanism of the brain findings.^14^

SARS‐CoV‐2 does not appear to be thrombogenic by itself. Instead, the coagulation abnormalities are secondary to the intense inflammatory reaction. The abnormal coagulation profile early in the infection is not translated to clinical bleeding compared with other RNA viruses causing hemorrhagic fevers.^24^

The risk of venous thromboembolism (VTE) in critically ill patients due to COVID‐19 is higher compared with the general ICU population, and the risk of VTE is still there even with prophylactic doses of anticoagulation.^24^, ^25^

Our patient had severe COVID‐19 pneumonia and ARDS that required prolonged mechanical ventilation with evidence of cytokine storm and hypercoagulability state. The course was complicated by corpus callosum hematoma, which is an uncommon site of ICH. Despite the improvement in the respiratory manifestation, the clinical picture became more complicated by developing pulmonary embolism.

In our patient, the endothelial dysfunction secondary to the intense inflammatory condition could explain the cerebral vessel damage that causes ICH rather than a manifestation of bleeding tendency, as the hypercoagulability state resulted in PE. COVID‐19 could be the umbrella under which all the events can be explained.

Reporting such a case might help increase the understanding of the neurological complications associated with COVID‐19 and increase awareness of possible challenges emerging while treating COVID‐19 patients.

## 4 CONCLUSION

Corpus callosum is a rare site for intracranial bleeding, complicating the course of severe COVID‐19 pneumonia.

## CONFLICT OF INTEREST

The authors report no conflict of interest.

## AUTHOR CONTRIBUTIONS

MKH involved in clinical care, literature review, and manuscript writing. DHT involved in literature review and manuscript writing. SNP involved in radiology imaging. MBH involved in manuscript writing VV involved in mentorship, clinical care, literature review, and manuscript revision.

## CONSENT

Written informed consent was obtained from the patient.

## Data Availability

The data that support the findings of this study are available from the author, MKH, upon reasonable request.
